# A computational view of area 3b of primary somatosensory cortex

**DOI:** 10.1186/1471-2202-14-S1-P333

**Published:** 2013-07-08

**Authors:** Georgios IS Detorakis, Nicolas P Rougier

**Affiliations:** 1 Computer Science, University of Lorraine, Nancy, 54000, France; 2 INRIA Nancy - Grand Est, Villers les Nancy, 54603 Cedex, France; 3 INRIA Bordeaux - Sud Ouest, Cours de la Liberation, 33405 Talence Cedex, France

## 

We investigated the development of topologically organized representations of a restricted region of skin in the primary somatosensory cortex (SI), more precisely, area 3b of SI. We devised a computational model based on the dynamic neural field theory and on an Oja-like learning rule at the level of feed-forward thalamocortical connections [1]. These connections reach area 3b through subthalamic and thalamic relays that convey information from the Merkel Ending Complexes (MECs), which are mechanoreceptors of the skin responsible for information related to touch and pressure. They have been modeled as a quasi-uniform grid while the rest of the relays have been neglected. Both the critical and the post-critical periods of the SI development [2] have been taken into consideration and the latter has been modeled as a long-term alteration of lateral connections. During the critical period, SI remains highly plastic and is able to cope with a vast number of alterations of the environment or of the body itself. This condition goes on during the post-critical period but in a less effective way [3]. In both periods SI is capable of reorganization in the presence of a cortical lesion [4] (e.g. stroke) or a sensory deprivation condition [5] (e.g. limb amputation). In order to examine if the model is capable of recovery from lesions, both cortical and sensory, we studied three different types of lesions on SI and on skin. As expected, the model is able to cope with such degenerative conditions and is able to recover a lot of the lost functionalities. More precisely, in the case of cortical lesions, neurons that are not affected can recover some of the lost representations while in the case of sensory deprivation, neurons that have lost their preferred input, tend to contribute to neighboring representations. Hence, the model confirms both cases and the mechanism of balance between excitation and inhibition seems to be the key for recovery. Attention is another aspect that has been investigated because of its prominent role in reshaping receptive fields during execution of demanding touch perception tasks [6]. In this context we simulated some attentional mechanisms in order to investigate how attention affects the receptive fields of the model. In the presence of an attentional signal, the model is able to gently adapt its receptive fields according to the position of the stimuli on the skin. On the one hand attention promotes the migration of the distant receptive fields towards the attended area and on the other hand proximal to attended signal receptive fields undergo shrinkage.
